# Stammering

**Published:** 1883-08

**Authors:** 


### STAMMERING.

TREATMENT OF STAMMERING.—Mr. J. E. Suitterlin has for eight years conducted an institute in this city for the cure of stuttering and stammering, with most satisfactory success. His system is philosophical and simple, and is based on the plainest common-sense principles. Excluding reliance on medical aids, it comprises chiefly careful drill of the vocal organs, and such mental discipline as will contribute to the object. In the first stage of treatment, the subject is not permitted to talk, except to practice his exercises, and to make such movements in speech as can be guided and observed by the teacher. During this time he is taught to consider himself not a patient, but a student of speech. In the second stage, which is begun when enough has been done in the first, the pupil is encouraged to talk, for practice, at every opportunity, with a "legato" movement (as in music) and a strong accent. In the third stage he is allowed to talk more naturally, but in a studied manner; and in the fourth stage he is permitted to employ his normal way of speaking, but is, by this time, relieved from the impediment under under which he formerly suffered. The psychic part of the treatment, which aims to divert the pupil's mind from himself and his troubles, is the most difficult and, at same time, the most essential part. The time required for success depends very largely and, in fact, chiefly on the mental constitution of the subject.

From this brief description of an effective method of treatment, the parent may gather the useful hint that, to remedy any incipient tendency in his child to stammer, he should exercise a mild and kind but firm ruling, suppress all irritability of temper, observe for the child all the laws of health, and be careful as to his own manner of talking and the patterns he may set for the child. By attention to such matters, even the most unskilled may correct the evil before the child begins to be conscious that he is a stammerer, and, by a general regard to such principles as are here laid down, the affliction might be wholly removed, or its frequency greatly reduced in the course of a generation or two. The statistics collected and preserved by Mr. Suitterlin show that the stammering habit is contracted, with only very rare exceptions, between infancy and ten years of age.—*The Popular Science Monthly* (*N. Y.*).

---

A NEW YORK chemist makes apple sauce out of chemicals. A surpassing triumph, however, awaits the man who can work over old Derby hats into charlotte russe.—*Lowell Citizen.*

